# Respiratory Health Symptoms among Students Exposed to Different Levels of Air Pollution in a Turkish City

**DOI:** 10.3390/ijerph8041110

**Published:** 2011-04-14

**Authors:** Hülya Gül, Eftade O. Gaga, Tuncay Döğeroğlu, Özlem Özden, Özkan Ayvaz, Sevda Özel, Günay Güngör

**Affiliations:** 1 Public Health Department, Istanbul Faculty of Medicine, Istanbul University, 34093, Çapa, Istanbul, Turkey; E-Mails: ozayvaz@istanbul.edu.tr (Ö.A.); ggungor@istanbul.edu.tr (G.G.); 2 Environmental Engineering Department, Faculty of Engineering and Architecture, Anadolu University, İki Eylül Campus, 26555, Eskişehir, Turkey; E-Mails: egaga@anadolu.edu.tr (E.O.G.); tdogeroglu@anadolu.edu.tr (T.D.); oozden@anadolu.edu.tr (Ö.Ö.); 3 Department of Biostatistics and Medical Informatics, Istanbul Faculty of Medicine, Istanbul University, 34390, Çapa, Istanbul, Turkey; E-Mail: sevda@istanbul.edu.tr (S.Ö.)

**Keywords:** air pollution, industry, public health, respiratory symptoms, student’s health

## Abstract

In this study, we aimed to investigate the frequency of respiratory health symptoms among high school students attending schools at industrial, urban and rural areas in a Turkish city. Three schools located in different zones of the city having different pollution characteristics were chosen based on the pollutant distribution maps using Geographical Information Systems (GIS) software. A cross-sectional survey was performed among 667 high school students in the schools. Outdoor and indoor nitrogen dioxide (NO_2_) and ozone (O_3_) concentrations were also measured by passive samplers in the same schools to investigate possible routes of exposure. Chronic pulmonary disease (OR = 1.49; 95%CI: 1.11–1.99; *p* = 0.008), tightness in the chest (OR = 1.57; 95%CI: 1.22–2.02; *p* = 0.001), morning cough (OR = 1.81 95%CI: 1.19–2.75; *p* = 0.006) were higher among students in the industrial zone where nitrogen dioxide and ozone levels were also highest. There were no indoor sources of nitrogen dioxide and ozone exists in the schools except for the dining hall. As a conclusion, this study has noticed that air pollution and respiratory health problems among high school students are high in industrial zones and the use of passive samplers combined with GIS is an effective tool that may be used by public health researchers to identify pollutant zones and persons at risk.

## 1. Introduction

Indoor and outdoor air pollution is one of the most serious environmental and public health problems in the industrialized world. Epidemiological evidence supports an association between exposure to ambient air pollutants (particulate matter (PM), nitrogen oxides (NO_x_), sulfur oxides (SO_x_), metals, volatile organics (VOCs), ozone (O_3_), *etc.*) and various health effects, such as respiratory symptoms or illness impaired cardiopulmonary function, reduction of lung function, and premature mortality [1–6]. Air pollution has more serious effects on high risk groups especially children, elderly people and individuals suffering from heart or lung diseases [7–11]. Although ambient air quality has important implications for health, indoor air quality is also a major concern since people spend much more time indoors. Exposure of adolescents to indoor air pollutants is mainly determined by concentrations of pollutants in three microenvironments: home, school and transport [12,13]. Major sources of pollutants measured indoors are derived from outdoor activities (traffic, industry, combustion, *etc.*), human activities inside (cooking, painting, cleaning, *etc.*), building equipment and furnishings. On the other hand, it is not easy to assess the air pollution status at fine spatial resolution in large geographical areas due to the prohibitive costs and manpower resources necessary for the measurement and monitoring of pollutants. Air pollutant concentrations are relatively high in densely populated congested locations in a city which means that exposure of people to those pollutants is expected to be higher compared to people living in less polluted locations. Preliminary information about pollutant spatial distribution in a geographical area is essential for identifying the risk to populations in a certain region. Geographical Information Systems (GIS) as a tool may provide help for the assessment of polluted and unpolluted sites by using pollutant concentrations measured at specific locations. Another point of interest in air pollution studies is the simultaneous measurement of pollutants at multiple locations by use of proper sampling devices. Passive samplers which are inexpensive, do not require electricity and easy to operate have been used for indoor and outdoor air quality assessment purposes [14,15]. The aim of the present study was to investigate the frequency of respiratory health symptoms among high school students exposed to different air pollution levels. The first step was the selection of the schools. For that purpose, a passive sampling campaign was carried out at nine locations to measure outdoor concentrations of NO_2_ and ozone in city. GIS was used to prepare pollution distribution maps for the city. Then, students were enrolled for questionnaire study. At the same period, indoor NO_2_ and ozone concentrations in different environments (classrooms, dining hall, library, *etc.*) and outdoor concentrations around school buildings were also measured to investigate whether school environment (indoor and outdoor) is a source for pollutant exposure of those students.

## 2. Materials and Methods

As the fifth most populated city in Turkey, Eskisehir (population: 724,849) is one of the cities with the highest educational level [16]. Coal usage in domestic heating has been gradually replaced with natural gas since 1996. Its topographical structure consists of plains surrounded by mountains. This cross-sectional study was conducted in high schools located in three different polluted zones of Eskisehir in June 2006. The design of the study can be summarized as follows:

- Preliminary assessment of the air pollution was made by using passive samplers.
- Schools were selected based on prepared distribution maps for NO_2_ and ozone with the help of ArcGIS software.
- A questionnaire was prepared and filled out via interviews with children.
- Questionnaire responses were evaluated together with outdoor and indoor pollutant concentrations.

### 2.1. Air Quality Parameters Analysis

**Figure 1.**
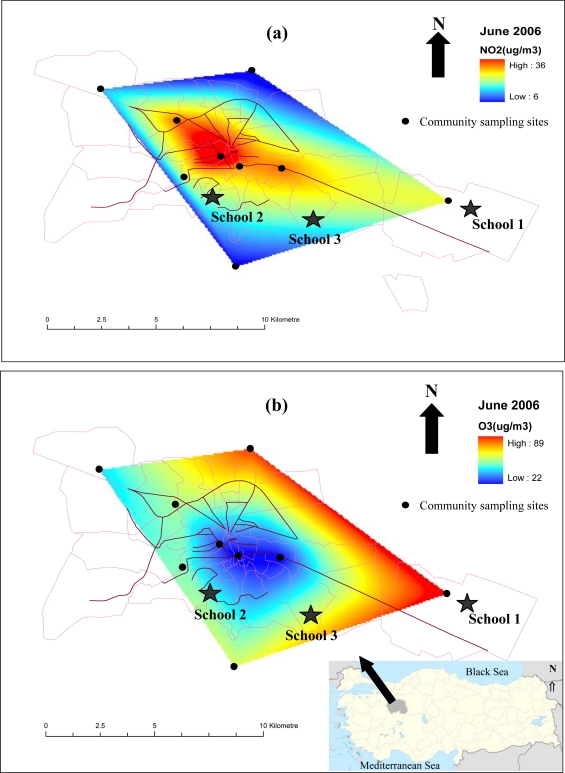
Spatial distribution of **(a)** NO_2_ and **(b)** ozone on June 2006.

Nitrogen dioxide (NO_2_) and ozone (O_3_) concentrations were measured at nine points throughout the city. Passive samplers were delivered at those points and collected back one week later so that totally four samples were collected for each location during month of June. Samples were analyzed and then measured average concentrations were used to prepare distribution maps of these pollutants (Figure 1) by using the ArcGIS 9.2 (ArcInfo) software. The distribution surfaces were created by using the “natural neighbor interpolation”. One of the schools (School 1) is located in the industrial area which is approximately 10 km east of the city centre. Small and medium sized industrial plants operate in this area dedicated to machine, metal, food and ceramic production. Natural gas is used for all power generation in this area. The second school (School 2) is within 100 m of a street and is located in the urban zone of the city having medium traffic density. The third school (School 3) is situated at one of the least polluted sites on the map, approximately 6 km south of the city centre in an area low in traffic density and far from major pollution sources. All the schools where this study was undertaken use natural gas in their heating systems.

Tailor made passive samplers were used to determine outdoor and indoor air quality in and around the schools. Two different types of passive samplers were used for the sampling. NO_2_ was collected in the Teflon passive sampler while O_3_ was collected in a Delrin passive sampler. The samplers have been derived from ANALYST^®^ type passive sampler. The samplers comprise a plastic body with the dimensions of 2.5 cm length and 2.0 cm inner diameter, filter paper, plastic ring, close plastic cap and stainless steel mesh barrier. For the preparation of NO_2_ passive samplers; Whatman GF/A fiberglass filter paper was impregnated with 20% TEA aqueous solution. For the preparation of ozone passive samplers; Whatman GF/A fiberglass filter paper was impregnated with 1% NaNO_2_ + 2% Na_2_CO_3_ + 2% glycerol aqueous solution. The filter papers were dried for a few minutes, placed to the bottom of the sampler and fixed with the 5 mm ring. The inlet ends were then closed with a plastic cap. After the sampling period, filter papers (for both blanks and exposed samplers) were transferred to the extraction vials and then extracted with 10 mL ultra pure distilled water for 15 min. The samples were analyzed using a DIONEX 2500 ion chromatography system.

Passive samplers were placed inside the schools in different environments (classroom, library, corridor, dining hall, teacher’s room) and outside the school building. A two-week sampling was carried out and after the sampling period, the samples were collected and then analyzed in the laboratory.

Accuracy of the NO_2_ passive sampler was determined by comparison with a Thermo 42i chemiluminescence NO-NO_2_-NO_x_ Continuous Automatic Gas Analyzer. 42 M UV Photometric Environment S.A. automatic ozone analyzer was used for the validation of ozone passive samplers. Routine calibrations of the automatic analyzers were carried out during field measurements. Percent relative error was found to be lower than 15% for both NO2 and ozone, indicating accuracy of the measurement. Precision of the method was described by coefficient of variation. Triplicate measurements of NO_2_ and ozone were carried out in the field to find precision of the samplers. Coefficient of variation was found lower than 11% for NO_2_ and approximately 12% for ozone. Detection limit of the method was determined by analyzing field blank samples. Three times of the standard deviation of field blanks set the detection limit and it was found to be 1.00 μg/m^3^ and 2.42 μg/m^3^ for a 1-week sampling period for NO2 and ozone, respectively.

### 2.2. Questionnaires

Each week one school was visited and all the questionnaires were completed in the month of June. Questionnaire forms were filled out during interviews with the students by the researchers. All students at each school filled out the questionnaires and those who had been living in the surrounding area of the schools they attend for a period at least 3 years were selected for the study. A total of 667 high school students from three schools participated in this cross-sectional survey. Written informed consent was obtained from the school directors and students.

The questionnaire was composed of two sections. The first set of questions focused on parents’ professions, education, number of rooms at home, heating type, household members, total monthly income, cigarette and alcohol habits of the students, family’s health history, *etc.* The second section consists of information about respiratory diseases and symptoms (physician-diagnosed chronic pulmonary disease, physician-diagnosed current asthma, physician-diagnosed bronchitis, persistent cough with phlegm, morning cough, morning phlegm, wheezing, chest tightness *etc.*) apart from cold and infection that was seen any time in the last 12 months. The questionnaire form was prepared based on the international studies and was tested in a pilot study before its use in this study [17–19].

### 2.3. Statistical Analysis

The statistical analysis was performed using the Statistical Package for the Social Sciences (SPSS) for Windows, version 16.0. Descriptive statistics were performed on the data set for all parameters. The Kolmogorov-Smirnov test was used for the fitness of the variables to the normal distribution. A value of *p* < 0.05 was considered statistically significant. The chi-square test (χ^2^) was used to examine differences among schools with respect to categorical variables. Non-smokers were defined as those who had never smoked any kind of tobacco. Smokers were those who were currently smoking at least one cigarette per day. Ex-smokers were those who had smoked previously and stopped more than one year ago. Passive smoking was defined as any current exposure to cigarettes, pipes or cigars in the home. Logistic regression analyses (Stepwise, Forward: LR) were used to estimate odds ratios (ORs) and the 95% confidence intervals (95% CIs). The regression models were tested using physician-diagnosed chronic pulmonary disease, wheezing, physician-diagnosed current asthma, tightness in the chest, physician-diagnosed bronchitis, persistent cough with phlegm, morning cough without infection and morning phlegm without infection as dependent variables. We also used gender, age, years of living at current address, education level of father and mother, job of father and mother, working status of the student, monthly income of family, sleeping in own room, passive smoking as independent variables. Logistic regression analysis was carried out for the parameters having p values smaller than 0.05 in the chi-square test primarily and the other variables which were considered important for clinically.

## 3. Results

In the present study, 667 high school students were enrolled, of which 545 were non-smokers. Table 1 shows the characteristics of the students. Two hundred and forty-nine (37.3%) of the students were living within few kilometres of the industrial organized region and attending school (School 1) in this area. Two hundred and fifty-four (38.1%) of the students were living within a few kilometres of School 2 in urban zone. One hundred and sixty-four (24.6%) of the students were living near School 3 in rural zone. The percentage of male students (68.2%) was higher than females (31.8%). The age distribution of the students was relatively homogeneous with 32.2% of 15 years old, 35.8% of 16 years old and 28.2% of 17 years old. Most of mothers were not employed in any kind of job (70.9%) and only 16.8% of mothers were working as white-collar workers. Regarding fathers’ occupation, 14.7% were white-collar workers and 19.2% were blue-collar workers. Based on parents’ monthly incomes, participants were assigned into groups as follows: less than 500, 500–2,000, and more than 2,000 United States Dollars per month for low, moderate and high-income groups, respectively. About 74.2% of the total monthly incomes of the families were moderate level. Most of the students (78.1%) had their own rooms and small fractions of them (2.2%) were working in a job.

The frequencies of self reported respiratory symptoms and diseases are given in Table 2. No statistically significant difference was found among the groups for wheezing, physician-diagnosed current asthma, physician-diagnosed bronchitis and persistent cough with phlegm. Statistically significant differences were found among groups for physician-diagnosed chronic pulmonary disease, tightness in the chest, morning cough and phlegm without infection.

Results of logistic regression models on the self reported respiratory symptoms and diseases of the students were presented in Table 3. After adjusted individual confounders, it was observed that odds ratios were high in industrial zone compared to rural zone for physician diagnosed chronic pulmonary disease, tightness in chest and morning cough without infection.

**Figure 2.**
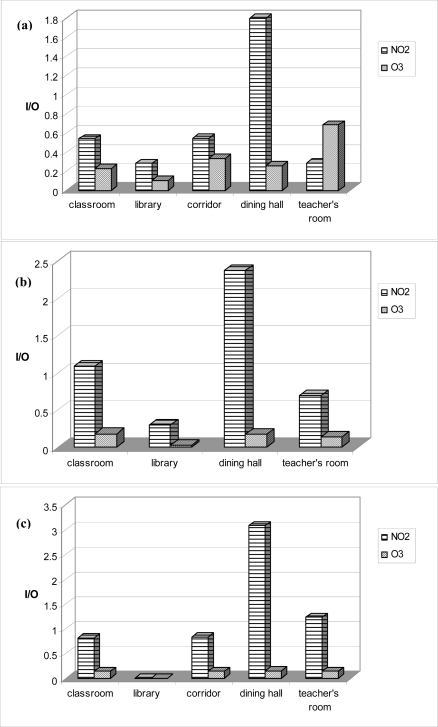
Average I/O ratios for each school for 2-week measurement period ((**a**) Industrial, (**b**) urban and (c) urban background schools in order of their appearance from top to bottom).

The outdoor NO_2_ concentration measured in School 1 was the highest (24.82 μg/m^3^) among the three sites, followed by School 2 (15.29 μg/m^3^) and School 3 (14.93 μg/m^3^), as expected from the distribution maps (Figure 1). The highest ozone concentration was measured in School 1 (83.05 μg/m^3^), followed by School 3 (75.45 μg/m^3^) and School 2 (60.12 μg/m^3^). I/O ratios calculated for each school were shown in Figure 2 and varied from 0.28–3.08 for NO_2_ and 0.03–0.68 for ozone in all schools.

## 4. Discussion

Exposure to air pollution from industrial and traffic sources is one of the most important public health problems. The intersection between air quality, student’s health and schools has also attracted the interest of many researchers and activists [20,21]. Adolescents may be particularly susceptible to the adverse effects of air pollution because they have a larger surface area and breathe more air per kilogram body weight than adults [22,23]. In this study, students living in industrial area showed higher rates of respiratory system symptoms (physician diagnosed chronic pulmonary disease, tightness in chest and morning cough without infection). These findings indicate that air pollution in the industrial areas is a risk factor in the prevalence of respiratory system symptoms and this is consistent with the results of other authors [24,25]. The findings of an Italian study suggest that emissions from chipboard industries might have a serious impact on children's respiratory health status [26]. Wilson *et al.* found the risks of respiratory symptoms in their study were increased by smoking, occupational exposures to dust and gas, and combined residence-related exposures such as living close to a main road, factory or chimney, indoor coal use and the presence of irritating smoke during cooking, among other risk factors [27]. Occurrence of CPD, wheezing and physician diagnosed asthma among adolescents whose family members have asthma and allergy was found to be higher than adolescents without asthma or allergy occurrence in their families. On the other hand, wheezing and physician diagnosed bronchitis was found to be higher among smokers and adolescents exposed to passive smoking respectively compared to non-smokers and not exposed to passive smoking. Cigarette abuse can be considered as an important environmental factor correlated with specified respiratory health complaints. In our study smoking was not very common among students with only a small percentage (12.6%) being smokers. Smoking in the workplace and in public places was common when this study was performed. It has been reported that 8.4% of 13–15 years old students and 34.6% of +18 years old adults are smokers in Turkey [28]. According to our results, statistically significant differences were observed among the three groups for health complaints such as chronic pulmonary disease, tightness in the chest, coughing and phlegm. Morgenstern *et al.* found that adjusted odds ratios (ORs) for wheezing, cough without infection, dry cough at night, bronchial asthma, bronchitis and respiratory infections indicated positive associations with traffic-related air pollutants [29]. They also found that increased levels of NO_2_ were associated with increased prevalence of respiratory health symptoms. Chen *et al.* mentioned that children living in the urban area had consistently higher rates of respiratory symptoms and diseases than did those living in the rural community [30]. According to results of the study of Langkulsen *et al.*, the prevalence of respiratory symptoms and impaired lung function were higher among children living in areas with high pollution than those in areas with low pollution [31]. Epton *et al.* detected no significant effect of ambient wood-smoke particulate air pollution on lung function of healthy school-aged male students, but a small effect on cough [32]. Small but significant effects of peak pollution levels were seen in students with asthma in their study. Arroya *et al.* (performed a study to estimate the impact of traffic flow on the prevalence of asthma among schoolchildren of 6 to7 and 13 to 14-years of age [33]. For both groups, the prevalence of asthma was found significantly related to traffic flow density. In our study no statistical difference was observed among the groups experiencing asthma. Among the three schools, 4.4% of the individuals from School 1, 2.0% from School 2, and 2.4% from School 3 experienced asthma. On the other hand, differences of respiratory symptoms were identified among adolescents regarding educational and job status of their parents. For instance, morning cough was found to be highest among adolescents whose mothers’ education level is low and this points needs to be investigated further.

With reference to Figure 1a, NO_2_ concentration around School 1 located in the industrial zone was found higher than the other two locations. This situation is largely related to the heavy traffic in the industrial area due to its proximity to major roads and big trucks carrying products in and out of the industrial organized region. The NO_2_ levels were lower in School 3, as it was far from traffic and any other kind of pollution source. As it is apparent from Figure 1b, high ozone concentrations were observed in places far from the city centre. Since ozone is a secondary pollutant, ozone levels are low in places close to the pollution sources and these levels increase as the distance from the pollution sources increases as in the case of School 1, which is far from the city centre. Several studies revealed that ozone was associated with increases in hospital admissions for asthma, school absenteeism for respiratory illnesses, respiratory problems associated with asthma and decreases in respiratory functions [34–36]. The relationship between NO_2_ and health effects including respiratory symptoms, episodes of respiratory illness, lung function and even mortality was shown in several studies [37,38].

Apart from the ambient air quality, air quality in and near schools is also important because students spend considerable amount of their time in the schools [39]. In this study, concentrations of NO_2_ and ozone were measured inside and outside the schools to understand their differing contributions to total exposure in the present study. According to Zhao *et al.* indoor chemical air pollutants of mainly outdoor origin could be risk factors for pupils’ respiratory symptoms at school [40]. The I/O ratios obtained at the schools were compared with a number of other studies in the literature carried out at schools with similar characteristics [41,42]. NO_2_ I/O ratio for S-1 (0.66) was a little lower than the literature values while the ratios of S-2 (1.05) and S-3 (1.17) were quite similar. None of the schools had air-conditioning systems and ventilation of classes was solely by opening windows. Significantly, I/O ratios for NO_2_ might be >1 in the indoor environments such as the dining hall or teacher’s room where cooking and smoking activities take place. I/O ratios of NO_2_ in dining halls where cooking activities take place were found to be highest in all three schools. In the majority of cases, the I/O ratios of NO_2_ were found to be close to or less than one. Low I/O values of ozone were also found in all schools indicating that no major source of ozone exists in indoor environments of the schools. High I/O ratios are an indication of indoor sources. It seems that classrooms are not a source of NO_2_ and ozone considering low I/O (<1) ratios measured. Indoor concentrations were mainly affected from outdoor concentrations for the classrooms where children spend significant amount of their times. Regarding outdoor concentrations measured around the schools, NO_2_ concentration was highest at the school located at industrial zone and ozone concentrations were also highest at this school. Outdoor air quality may affect respiratory health symptoms of those children. The results of this study suggest that air quality in industrially polluted sites might increase the risk of respiratory health conditions of students. We found out that the frequency of the indicators related to some measures of respiratory health was higher for the high school students in the industrial zone than to those in the urban and rural zones.

On the other hand, a much more comprehensive study is required to apportion contribution of ambient air quality at schools and living environments to respiratory health status of the children. One of the major limitations of this study is the self-reported respiratory outcomes because of recall error *etc.* Another important limitation is the use of short-term monitoring to represent long-term exposures. Children are exposed to air pollutants mainly in three microenvironments: school, home and transportation. In this study, concentrations of certain air pollutants at school environments were measured so that we can’t assess the contribution of pollution at home and during transportation.

Measurement of other pollutants such as SO_2_ and PM might provide more information about exposure amounts and pathways of the pollutants. This study is a preliminary and descriptive work that leads the way to more extensive studies in this region. For the future studies, other important pollutants such as SO_2_ and PM are planned to be included in the air quality measurements. Besides indoor and outdoor air quality measurements in the schools, determination of personal exposures of the students and also air quality measurements in and around their houses should also be included in the future studies.

From public health perspective, it is important to control the possible risks on the health of the students. By considering the fact that young individuals are much more sensitive to the contaminating effects of air pollution, regulatory authorities should deal with this topic seriously. Geographical Information Systems (GIS) can be used by public health researchers as a tool for the identification of polluted zones and populations at risk in a certain geographical area. Passive samplers which are easy to use, economical and require minimal manpower are suitable for the simultaneous measurement of pollutants at many locations.

## Figures and Tables

**Table 1.** Characteristics of the study subjects (*n* = 667).

**Variable**	**Total**		**School 1 (*n* = 249)**		**School 2 (*n* = 254)**		**School 3 (*n* = 164)**	

	***n***	**%**	***n***	**%**	***n***	**%**	***n***	**%**
**Gender**								
Male	455	68.2	192	77.1	169	66.5	94	57.3
Female	212	31.8	57	22.9	85	33.5	70	42.7

**Age (year)**								
15	215	32.2	81	32.5	74	29.1	60	36.6
16	239	35.8	92	36.9	81	31.9	66	40.2
17	188	28.2	70	28.1	83	32.7	35	21.3
18	25	3.7	6	2.4	16	6.3	3	1.8

**Living at current address (year)**									
3–5	420	62.9	169	67.9	190	74.8	61	37.2
6–10	98	14.7	28	11.2	30	11.8	40	24.4
11–15	121	18.2	36	14.5	32	12.6	53	32.3
≥16	28	4.2	16	6	2	0.8	10	6.1

**Mother’s education**								
≤Primary school	250	37.5	134	53.8	60	23.6	56	34.1
Middle school	103	15.4	55	22.1	24	9.5	24	14.6
High school	162	24.3	42	16.9	68	26.8	52	31.7
University	152	22.8	18	7.2	102	40.1	32	19.6

**Father’s education**								
≤Primary school	118	17.7	75	30.1	14	5.5	29	17.6
Middle school	82	12.3	57	22.9	13	5.1	12	7.3
High school	204	30.6	82	32.9	65	25.6	57	34.8
University	263	39.4	35	14.1	162	63.8	66	40.3

**Mother’s job**								
Housewife	473	70.9	221	88.8	136	53.5	116	70.7
Retired	52	7.8	7	2.8	32	12.6	13	7.9
Blue collar worker	14	2.1	7	2.8	0	0	7	4.3
White collar worker	112	16.8	14	5.6	81	31.9	17	10.4
Other	16	2.4	0	0	5	2.0	11	6.7

**Father’s job**								
Retired	126	18.9	39	15.7	48	18.9	39	23.8
Blue collar worker	128	19.2	83	33.3	19	7.5	26	15.9
White collar worker	98	14.7	51	20.5	27	10.6	20	12.2
Own work	25	3.7	12	4.8	11	4.3	2	1.2
Farmer	175	26.2	48	19.3	95	37.4	32	19.5
Driver	17	2.5	12	4.8	1	10.4	4	2.4
Other	98	14.6	4	1.6	53	20.9	41	25.0

**Student**								
Yes works	14	2.1	7	2.8	0	0.0	7	2.3
No doesn’t work	653	97.9	242	97.2	254	100	157	95.7

**Monthly income**								
Low	68	10.0	56	22.5	4	1.6	8	4.9
Medium	494	74.2	186	74.7	178	70.1	130	79.3
High	105	15.7	7	2.8	72	28.3	26	15.9

**Own room**								
There is	521	78.1	198	79.5	181	71.3	142	86.6
There is not	146	21.9	51	20.5	73	28.7	22	13.4

**Smoking status**								
Current smoker	84	12.6	61	24.5	7	2.8	16	9.8
Never smoked	545	81.7	168	67.5	233	91.7	144	87.8
Ex smoker	38	5.7	20	8.0	14	5.5	4	2.4

**Passive smoker**								
Yes	370	55.5	92	36.9	159	62.6	119	72.6
No	297	44.5	157	63.1	95	37.4	45	27.4

School 1: industrial zone; School 2: urban zone; School 3: rural zone.

**Table 2.** Distribution of respiratory symptoms among high school students (*n* = 667).

**Symptom**	**Total**		**School 1 (*n* = 249)**		**School 2 (*n* = 254)**		**School 3 (*n* = 164)**		**Chi-square**	**(*p*)**

	***n***	**%**	***n***	**%**	***n***	**%**	***n***	**%**		
**Physician diagnosed chronic pulmonary disease**										
Yes	105	15.7	54	21.7	30	11.8	21	12.8	10.660	0.005 *
No	562	84.1	195	78.3	224	88.2	143	87.2		
Total	667	100	249	100	254	100	164	100		

**Wheezing**										
Yes	126	19.0	57	22.9	43	16.9	26	15.9	4.226	0.121
No	541	81.0	192	77.1	211	83.1	138	84.1		
Total	667	100	249	100	254	100	164	100		

**Physician-diagnosed current asthma**										
Yes	20	3.0	11	4.4	5	2.0	4	2.4	2.827	0.243
No	647	97.0	238	95.6	249	98.0	160	97.6		
Total	667	100	249	100	254	100	164	100		

**Tightness in the chest**										
Yes	157	23.5	77	30.9	48	18.9	32	19.5	12.063	0.002 *
No	510	76.5	172	69.1	206	81.1	132	80.5		
Total	667	100	249	100	254	100	164	100		

**Physician diagnosed bronchitis**										
Yes	187	28.0	80	32.1	61	24.0	46	28.0	4.102	0.129
No	480	72.0	169	67.9	193	76.0	118	72.0		
Total	667	100	249	100	254	100	164	100		

**Persistent cough with phlegm**										
Yes	336	50.4	114	45.8	128	50.4	94	57.3	5.262	0.072
No	331	49.6	135	54.2	126	49.6	70	42.7		
Total	667	100	249	100	254	100	164	100		

**Morning cough without infection**										
Yes	62	9.3	36	14.5	15	5.9	11	6.7	12.635	0.002 *
No	605	90.7	213	85.5	239	94.1	153	93.3		
Total	667	100	249	100	254	100	164	100		

**Morning phlegm without infection**										
Yes	59	8.8	32	12.9	16	6.3	11	6.7	7.928	0.019 *
No	608	91.2	217	87.1	238	93.7	153	93.3		
Total	667	100	249	100	254	100	164	100		

* There is a statistical significance.

**Table 3.** Results of logistic regression models on respiratory symptoms of students * (*n* = 667).

**Dependent variables with only significant risk factors (*p* < 0.05)**	**Model coefficient (B)**	**Standard error**	**Statistical significance (*p*)**	**Odds Ratio (95%CI: lower-upper)**
**Physician diagnosed chronic pulmonary disease**				
Industrial zone (ref = school 3 )	0.396	0.150	0.008	1.49 (1.11–1.99)
In family asthma, allergy etc (ref = no )	0.927	0.239	0.0001	2.53 (1.58–4.04)
Gender (ref = female)	0.569	0.260	0.029	1.77 (1.06–2.94)

**Wheezing**				
In family asthma, allergy etc (ref = no )	0.547	0.240	0.022	1.73 (1.08–2.76)
Mother’s job (ref = housewife)	–0.999	0.321	0.002	0.37 (1.01–3.16)
Smoking (ref = no)	0.521	0.208	0.012	1.68 (1.12–2.53)

**Physician –diagnosed current asthma**				
In family asthma, allergy *etc.* (ref = no )	1.344	0.465	0.004	3.80 (1.53–9.45)
Working status of student (ref = no )	2.168	0.721	0.003	8.74 (2.13–35.92)

**Tightness in the chest**				
Industrial zone (ref = school 3 )	0.450	0.130	0.001	1.57 (1.22–2.02)

**Physician –diagnosed bronchitis**				
Passive smoker (ref = no )	0.465	0.188	0.013	1.59 (1.10–2.30)

**Persistent cough with phlegm**				
Father’s job (ref = retired)	0.586	0.213	0.006	1.79 (1.18–2.72)

**Morning cough without infection**				
Industrial zone (ref = school 3 )	0.592	0.215	0.006	1.81 (1.19–2.75)
Mother’s education (ref = middle school)	–0.650	0.281	0.021	0.52 (0.30–0.90)

**Morning phlegm without infection**				
Smoking (ref = no)	0.581	0.235	0.014	1.79 (1.13–2.83)

ref = reference;

* = All the independent variables in Table 1 and school zones were included in the models.
